# In-depth study of personality disorders in first-admission patients with substance use disorders

**DOI:** 10.1186/1471-244X-12-180

**Published:** 2012-10-29

**Authors:** Anne-Marit Langås, Ulrik Fredrik Malt, Stein Opjordsmoen

**Affiliations:** 1 Vestre Viken Hospital Trust, Division of Mental Health and Addiction, Department of Mental Health Research and Development, P.O. Box 135, Lier, NO-3401, Norway; 2 Norwegian Research Network on Mood Disorders (NORMOOD), Oslo, Norway; 3 University of Oslo, Institute of Clinical Medicine, Oslo, Norway; 4 Division of Surgery and Clinical Neuroscience, Dept of Neuropsychiatry and Psychosomatic Medicine, Oslo University Hospital, Oslo, Norway; 5 Division of Mental Health and Addiction, Dept of Research and Development, Oslo University Hospital, Oslo, Norway

**Keywords:** Substance use disorder, Personality disorder, Comorbidity, Treatment-naïve, Catchment area

## Abstract

**Background:**

Assessment of comorbid personality disorders (PDs) in patients with substance use disorders (SUDs) is challenging due to symptom overlap, additional mental and physical disorders, and limitations of the assessment methods. Our in-depth study applied methods to overcome these difficulties.

**Method:**

A complete catchment area sample of 61 consecutively admitted patients with SUDs, with no previous history of specialized treatment (addiction clinics, psychiatry) were studied, addressing PDs and associated clinical and demographic variables. The thorough assessments included the Psychiatric Research Interview for Substance and Mental Disorders and the Structured Clinical Interview for DSM-IV Axis II Personality Disorders.

**Results:**

Forty-six percent of the SUD patients had at least one PD (16% antisocial [males only]; 13% borderline; and 8% paranoid, avoidant, and obsessive-compulsive, respectively). Cluster C disorders were as prevalent as Cluster B disorders. SUD patients with PDs were younger at the onset of their first SUD and at admission; used more illicit drugs; had more anxiety disorders, particularly social phobia; had more severe depressive symptoms; were more distressed; and less often attended work or school.

**Conclusion:**

The psychiatric comorbidity and symptom load of SUD patients with PDs differed from those of SUD patients without PDs, suggesting different treatment needs, and stressing the value of the assessment of PDs in SUD patients.

## Background

Personality disorders (PDs) are among the most prevalent comorbid disorders in treatment-seeking patients with substance use disorders (SUDs). Studies of outpatients with alcohol use disorders (AUDs) have found a prevalence of PDs between 40% and 64% [1-4]. In samples of inpatients with AUD, the prevalence ranges from 34% to 78% [5-7]. In studies that include drug use disorders (DUDs), the prevalence has been found to be between 35% and 91% [7-13]. Studies suggest antisocial PD and borderline PD to be the most prevalent PDs in SUD subjects by a wide margin, comprising up to 33.5% and 27.7% of samples, respectively [13]. Therefore, many studies have only included the assessment of antisocial PD or antisocial and borderline PDs. However, some studies have found other PDs to be more prevalent. In a sample of inpatients with AUD, Preuss et al. found that obsessive-compulsive, borderline, narcissistic, and paranoid disorders, in that order, were the most prevalent PDs [6]. In a sample of AUD outpatients, Echeburua et al. found obsessive-compulsive, antisocial, paranoid, and dependent PDs to be the most prevalent [3]. In another sample of AUD outpatients, borderline PD, PD not otherwise specified (NOS), narcissistic PD, and obsessive-compulsive PD were the most prevalent [4]. In DeJong et al.’s study, the order of PDs with the highest prevalence in AUD patients was histrionic, dependent, avoidant, and compulsive, while the order in DUD patients was borderline, histrionic, passive-aggressive, and antisocial [7].

As substance use, mental disorders, and physical diseases have many overlapping symptoms, it is difficult to decide to which disorder a symptom should be attributed in patients with multiple disorders. Reliable PD diagnoses in SUD patients can only be achieved when the interviewer is aware of the comorbid disorders and has the necessary clinical skills to disentangle the disorders from each other, preferably guided by an interview designed for this purpose. A symptom should not be attributed to a PD if it only occurs during heavy substance use, withdrawal, or an active phase of an Axis I disorder. In addition, the Diagnostic and Statistical Manual of Mental Disorders, Fourth Edition, Text Revision [14] states that a PD should not be based “…solely on behaviors that are consequences of Substance Intoxication or Withdrawal or that are associated with activities in the service of sustaining a dependency (e.g., antisocial behavior)” (DSM-IV-TR page 688–689). Some personality changes, such as antisocial or paranoid features, are common after long-term heavy substance abuse. In contrast to the DSM-IV, the World Health Organization’s ICD-10 Classification of Mental and Behavioral Disorders, includes the possibility of a substance-induced change in personality [15].

The association between SUDs and PDs is not simply the result of diagnostic methodology or symptom overlap. Verheul et al. found that PDs did not remit with SUD remission after SUD treatment [16]. Rounsaville et al. differentiated between primary and substance-induced symptoms of PD diagnoses, but the prevalence of PD diagnoses without substance-related symptoms was still high [9]. Skodol et al. found that the prevalence of PD was as high in patients with past SUDs as in patients with present SUDs [17].

It is important to clarify any differences between SUD patients with PDs and those without. Studies suggest that SUD patients with PDs are younger, have lower levels of education, are less likely to be married, are more likely to abuse illegal substances, have a different pattern of alcohol use, have more psychopathology, including anxiety and depression, are more impulsive, and are less satisfied with life [12,18]. One study, however, found no substantial differences apart from more anxiety and depression in the SUD patients with PDs [10].

In conclusion, previous studies of PDs in SUD patients have some limitations: the selection of convenience samples, the mixture of patients with different treatment histories, the use of lay interviewers, and the application of diagnostic interviews of nonoptimal validity and reliability in comorbid SUD, Axis I, or Axis II disorders. No studies based on catchment-area samples of patients admitted for the first time have been published.

Norwegian psychiatric and addiction services are public, based on catchment areas and are available to everyone. Almost all patients with mental or addiction problems are primarily referred to the psychiatric department of the local hospital for their catchment area. This allowed us to study a sample of all patients with SUDs consecutively admitted to specialist psychiatric or addiction services for the first time within a specified time period from one catchment area.

Our null hypotheses were: 1) The only prevalent and clinically relevant PDs in SUD patients entering treatment for the first time are antisocial PD and borderline PD, and 2) there are no differences between first-time admitted SUD patients with and without PDs in demographics, comorbidity, symptoms, or functioning.

## Methods

### Participants

All patients with SUDs from the catchment area who were 16 years and older, had no previous history of adult specialized addiction or psychiatric treatment, and were consecutively admitted to inpatient or outpatient treatment within a period of 18 months were included.

The catchment area was a rural area in southeastern Norway. The main town is situated 82 km from Norway’s capital, Oslo. All commonly used legal and illegal substances were available. Patients referred for addiction treatment were presumed to have an SUD and were asked to give their consent to participate in the study. Patients referred for psychiatric treatment were screened for substance use problems using the Alcohol Use Disorders Identification Test (AUDIT) [19] and the Drug Use Disorders Identification Test (DUDIT) [20]. The chosen cutoffs were scores of six (females) and eight (males) for the AUDIT, and two (females) and six (males) for the DUDIT. Patients who scored above the cutoff were asked for their consent and referred to the study.

The included patients were required to be cooperative in the study. All patients were assessed in a stable state of their SUDs and other Axis I disorders, so that Axis I symptoms did not interfere with the Axis II judgment. Because most SUD patients in Norway are assessed as outpatients before they are referred to inpatient treatment, most of the sample patients were outpatients. Only five were inpatients, and three of these were inpatients only during parts of their assessment period.

Of the 93 patients identified by their therapists as fulfilling the inclusion criteria, 78 gave their written consent to take part in the study. Of the 15 patients who refused to take part, six were male and nine were female, their mean age was 30.1 years, and all had alcohol as their main substance of abuse. In addition, one patient abused cannabis and one patient abused sedatives. Of the 78 patients who gave their consent, four dropped out. Of these four, three were male and one was female, and their mean age was 23.8 years. Three of them had polysubstance dependence. Data for the fourth were not available. Altogether, 74 patients accomplished all or most of the assessments, but of these, one gave unreliable data because of an organic brain disorder. Of the 73 included patients, 12 (16.4%) did not fulfill the diagnostic criteria for any SUD diagnosis (that is, dependence or abuse) despite positive screening. The analyses presented here were carried out on the 61 patients with one or more SUD diagnoses.

### Measurements

Demographics, family history of mental disorders and SUDs, and basic data about general health were registered with the shortened version of the Stanley Foundation’s Network Entry Questionnaire (NEQ) [21]. We collected information about physical health, as well as extensive blood samples, to test for somatic causes of the symptoms.

The substance use history, SUDs, other Axis I disorders, age at onset, and time periods where the diagnostic criteria for a disorder were fulfilled were assessed with the Psychiatric Research Interview for Substance and Mental Disorders (PRISM) for DSM-IV [22,23]. This interview was designed in accordance with the DSM-IV criteria for differentiating between independent and substance-induced disorders, and includes instructions for the identification and exclusion of expected effects of substance intoxication or withdrawal. A clinically experienced psychiatrist (first author), who had attended PRISM training arranged by the group at Columbia University, where the interview was developed, performed all the PRISM interviews.

Personality disorders were assessed with the Structured Clinical Interview for DSM-IV Axis II Personality Disorders (SCID-II). The SCID-II is an instrument with good reliability for diagnosing PDs [24]. A psychiatrist experienced in SUD and PD diagnostics, and formally trained in reliable and valid SCID-II interviewing at the Department of Personality Psychiatry, Oslo University Hospital (first author), performed all SCID-II interviews. The SCID-II interview was performed after the PRISM, and the interviewer was aware of the patients’ Axis I disorders. Throughout the SCID-II interview, each patient was reminded several times not to include symptoms restricted to periods of heavy substance use, withdrawal, or other Axis I disorders.

The categorical PD diagnoses, based on a specific number of criteria for each diagnosis, have some limitations. The Personality and Personality Disorders Work Group for the DSM-5 has recommended a significant reformulation of the approach to the assessment and diagnosis of personality psychopathology: a hybrid dimensional-categorical model [25]. These recommendations were not available at the time of our data collection. Therefore, we included the group of PD patients with subthreshold diagnoses in the PD diagnosis group in our analyses. We defined subthreshold as one criterion short of the diagnostic criteria for each diagnosis according to the DSM-IV. By including the subthreshold cases, we were able to explore whether the severity or dimensional aspect of PDs, measured by the number of criteria, influenced the results. The PDs described in Appendix B of the DSM-IV (depressive PD and passive-aggressive [negativistic] PD) were not included in this study.

The Global Assessment of Functioning (GAF) split version was used for the overall assessment of symptoms and functioning. The first author carried out the GAF assessment. She had taken part in several GAF rating exercises with experienced raters. Previous studies of the reliability of the GAF indicate that GAF scores assigned by raters in research situations are reliable provided there is sufficient interrater training [26]. Both symptom and function scores of the GAF were consistent across experienced raters [27].

We applied the revised version of the 90-question Symptom Checklist (SCL-90-R) [28] as a screening instrument for psychiatric symptoms. Studies have shown that the SCL-90 has high sensitivity and moderate specificity when used as a screening instrument for mental disorders in SUD patients [29,30]. The sum score, the Global Severity Index, can be used as a measure of overall psychological distress. The nine subscales may not be considered to reflect separate independent dimensions [31].

Depressive symptoms were further investigated with the Inventory of Depressive Symptoms (IDS) [32]. The IDS interviews were performed by the first author, who had attended reliability training with the following results: ICC = 0.97 [95% CI: 0.87–0.99], or a nurse specialized in clinical psychiatry. She underwent a training course and completed the first three interviews under the supervision of the first author.

We did not divide the SUD diagnoses into ‘abuse’ or ‘dependence’ for analysis, in accordance with the suggestion by the DSM-5 Work Group [25]. AUD and DUD are the subgroups of SUDs included in our study, where DUD covers abuse of or dependence on illicit drugs or prescribed medications with misuse potential. We use the terms ‘AUD only’ to refer to AUD without DUD, and ‘DUD only’ to refer to DUD without AUD.

The age at onset of a disorder was defined as the age at which the patient met the full criteria of the diagnosis. The duration of an untreated disorder was defined as the sum of all periods in which the patient met the full criteria of the disorder until first admission to specialized health services.

More details of the methods have been described in an earlier publication [33]. The study received approval from the South-Eastern Norway Regional Committee for Medical Ethics (REC, reference number 109-08072d 6.2008.100), and from the Norwegian Social Science Data Services. The study complies with the Helsinki Declaration.

### Statistical analysis

SPSS software (PASW® Statistics version 16.0; IBM, Armonk, NY, USA), was used for the analyses. Statistical significance was determined using the 0.05 level and two-tailed tests of significance. Chi-square tests with continuity correction or Fisher’s exact tests were used to investigate group differences on categorical data. Independent sample *t*-tests were used to compare groups on normally distributed continuous variables. Mann–Whitney *U* tests were used to compare groups on continuous variables that were not normally distributed.

## Results

### Demographics

The demographic data for SUD patients with and without comorbid PDs are shown in Table 1. One-third of the sample was female, with no statistically significant difference between groups. SUD patients with PDs had a lower age at admission and less education, and attended school or work less often than SUD patients without PDs.

**Table 1:** Demographics in first-admission patients with substance use disorders, with and without personality disorders

	**PD full criteria**			**PD including subthreshold diagnoses**		
	**Yes**	**No**	**Sign.**	**Yes**	**No**	**Sign.**
	***n *= 28**	***n = *33**		***n *= 35**	***n = *26**	
**Demographics**						
Age at admission (mean years)	27.4	34.9	*	27.4	36.8	*
Female (%)	29	36		29	39	
Education (mean years)	11.2	12.9	***	11.7	12.8	*
Married/cohabiting (%)	39	24		37	23	
Work or school (%)	21	61	**	29	62	*

*Notes.* * p < 0.05; ** p < 0.01; *** p ≤ 0.001.

*PD* personality disorder, *Subthreshold* lacking one criterion for the diagnosis, *Sign.* statistically significant differences between patients with and without PD.

### Prevalence of PDs

Of the 61 patients with one or more lifetime SUDs, 28 (46%) had one or more PD diagnoses: 21 had one PD, three had two PDs, three had three PDs, and one had four PDs. Table 2 shows the prevalence of the different PDs and the total numbers within clusters. We found numerically more patients in Cluster B (21%) than Cluster C (18%) or Cluster A (8%).

**Table 2:** Prevalence of different personality disorders in patients admitted for the first time with substance use disorders

	**PD full criteria**	**PD including subthreshold diagnoses**
	***n *(%)**	***n *(%)**
*Any Cluster A*	*5 (8)*	*12 (20)*
Paranoid	5 (8)	11 (18)
Schizoid	0	1 (2)
Schizotypal	1 (2)	1 (2)
*Any Cluster B*	*13 (21)*	*17 (28)*
Antisocial	10 (16)	12 (20)
Borderline	8 (13)	10 (16)
Histrionic	0	1 (2)
Narcissistic	0	0
*Any Cluster C*	*11 (18)*	*19 (31)*
Avoidant	5 (8)	8 (13)
Dependent	1 (2)	4 (7)
Obsessive-compulsive	5 (8)	12 (20)
*PD NOS*	5 (8)	na
*Any PD*	*28 (46)*	*35 (57)*

*Notes. Subthreshold diagnoses* lacking one criterion for the diagnosis. Some patients have more than one PD within the same cluster. Some patients have PDs from more than one cluster; *NOS* not otherwise specified, *na* not applicable.

Antisocial PD (16%) and borderline PD (13%) were the most prevalent disorders. Paranoid, avoidant, obsessive-compulsive, and PD NOS were equally prevalent (8% each). Schizotypal and dependent PD were found in one patient each. None of the patients in our sample met the full criteria for schizoid, narcissistic, or histrionic PD. Table 2 also shows the prevalence of disorders when subthreshold diagnoses were included. We found that 57% of the patients had a PD or subthreshold PD, with more patients in Cluster C than in Cluster B. Obsessive-compulsive PD was as prevalent as antisocial PD (20%).

Comparison of the prevalence of PDs in male and female patients showed a significant difference only in antisocial PD. None of the female patients had antisocial PD, but 24% of the males did (p = 0.023). When we compared AUD-only patients with patients who had DUD with or without additional AUD, we found significantly more PDs in the DUD group (74% versus 29%, p = 0.025).

### SUDs

Of the total sample, 53 patients (87%) had AUD and 33 patients (54%) had DUD. Twenty-eight patients (46%) had AUD only and eight patients (13%) had DUD only. Twenty-five patients (41%) had both AUD and DUD. The 33 patients with DUD abused or were dependent on the following substances (number of patients in parentheses): cannabis (28), sedatives (13), stimulants (10), cocaine (9), opioids (2), and other substances (2). Of the polysubstance users, 10 patients had two SUDs, 10 patients had three SUDs, three patients had four SUDs, and four patients had five SUDs.

Table 3 shows the different SUDs in SUD patients with and without PDs. Patients without PDs more often had AUD only, while patients with PDs more often had both AUD and DUD. The higher DUDIT score in SUD patients with PDs also confirmed the more prevalent use of illicit drugs, especially cannabis and stimulants. The PD group smoked about twice as many nicotine cigarettes per day on average. These patients also showed other signs of more serious substance use problems; they had an earlier age of onset of their first SUD, longer total duration of SUDs before admission to treatment, and a higher number of SUD diagnoses.

**Table 3:** Substance use disorders and other substance use assessments in patients with and without comorbid personality disorders

	**PD full criteria**			**PD including subthreshold diagnoses**		
	**Yes**	**No**	**Sign.**	**Yes**	**No**	**Sign.**
	***n *= 28**	***n = *33**		***n *= 35**	***n = *26**	
**Substance use disorders**						
AUD only (%)	29	61	*	34	62	
DUD only (%)	11	15		9	19	
AUD and DUD (%)	61	24	**	57	19	**
Sedatives (%)	29	15		29	12	
Cannabis (%)	64	30	*	57	31	
Stimulants (%)	29	6	*	26	4	*
Cocaine (%)	21	9		20	8	
Opioids (%)	7	0		6	0	
Cigarettes per day (mean number)	12.6	6.7	**	11.0	7.3	
**SUD characteristics**						
Age first SUD (mean years)	18.5	27.9	***	19.8	28.6	**
Total duration of SUD (mean years)	8.4	5.0	*	7.2	5.7	
Number of SUDs (mean)	2.4	1.5	**	2.3	1.4	**
AUDIT sum score (mean)	13.3	15.1		13.3	15.5	
DUDIT sum score (mean)	14.2	8.8	*	13.3	8.5	

*Notes.* All diagnoses are lifetime diagnoses. * p < 0.05; ** p < 0.01; *** p ≤ 0.001.

*PD* personality disorder, *Subthreshold* lacking one criterion for the diagnosis, *SUD* substance use disorder, *AUD* alcohol use disorder, *DUD* drug use disorder, *AUDIT* Alcohol Use Disorders Identification Test, *DUDIT* Drug Use Disorders Identification Test, *Sign*. statistically significant differences between patients with and without PD.

### Axis I comorbidity

Of the 61 patients with SUD diagnosis, 11.5% had no comorbid Axis I or Axis II disorders, 42.6% had a comorbid Axis I disorder, and the same percentage had both Axis I and Axis II comorbidity. Only two patients had an Axis II disorder without a comorbid Axis I disorder. Comorbid Axis I disorders were highly prevalent in SUD patients with and without PDs (Table 4). Only the most prevalent affective and anxiety disorders are specified in Table 4. We found a significantly higher mean number of lifetime Axis I disorders in the PD group. Of the different anxiety disorders, social phobia occurred significantly more often in PD patients. There was no difference in the prevalence of mood disorders; however, the age at onset of any mood disorder was significantly lower in SUD patients with PDs than in SUD patients without PDs (17.4 years compared to 26.3 years; p = 0.027). The distribution of independent and substance-induced major depressive disorder (MDD) did not differ between groups.

**Table 4:** Comorbidity and symptom assessments in patients with substance use disorders with and without comorbid personality disorders

	**PD full criteria**			**PD including subthreshold diagnoses**		
	**Yes**	**No**	**Sign.**	**Yes**	**No**	**Sign.**
	***n *= 28**	***n = *33**		***n *= 35**	***n = *26**	
**Comorbid Axis I disorders**						
Any Axis I disorder^a^ (%)	93	79		91	77	
Number of Axis I disorders^a^ (mean)	2.6	1.7	*	2.5	1.7	
Any mood disorder (%)	79	73		80	69	
MDD (%)	68	70		69	69	
Any anxiety disorder (%)	68	36	*	60	39	
Social phobia (%)	50	15	**	40	19	
Panic disorder (%)	18	6		14	8	
PTSD (%)	21	15		17	19	
**Other assessments**						
SCL-90-R, GSI score (mean)	1.45	0.94	*	1.37	0.92	*
IDS sum score (mean)	30.5	22.4	**	28.0	23.6	
GAF-S (mean score)	51.4	58.8	***	53.0	58.7	**
GAF-F (mean score)	49.5	61.4	***	51.5	61.9	***

*Notes.* All diagnoses are lifetime diagnoses. ^a^ Axis I disorders other than SUDs. * p < 0.05; ** p < 0.01; *** p ≤ 0.001.

*PD* personality disorder, *Subthreshold* lacking one criterion for the diagnosis, *SUD* substance use disorder, *MDD* major depressive disorder, *PTSD*, post-traumatic stress disorder, *GAF-S* Global Assessment of Functioning, Symptom score, *GAF-F* Global Assessment of Functioning, Functioning score, *SCL-90-R* Symptom Checklist, 90 Questions, Revised, *GSI* Global Symptom Index, *IDS* Inventory of Depressive Symptoms, *Sign.* statistically significant differences between patients with and without PD.

### Other assessments

Patients with PDs had a higher symptom load as measured by the SCL-90-R. There were statistically significant differences on all the subscales except Depression, Somatization, and Additional (mainly measuring problems with sleep or appetite). The main differences were found on the Paranoid Ideation, Hostility, Psychoticism, and Phobic Anxiety subscales, and were also statistically significant after Bonferroni correction. The patients with comorbid PDs were more depressed, according to the IDS, and had more severe symptoms and poorer functioning, according to the GAF scores.

The mean age at onset of the different disorders was low in patients with PDs. Patients with anxiety disorders had a mean age at onset of nine years, the first SUD had a mean age at onset of 17 years, and the first affective disorder occurred at a mean age of 18 years.

We repeated the analyses with the subthreshold diagnoses included in the PD group (as shown in Tables 3 and 4). This caused a greater increase in the prevalence of some of the PDs (obsessive-compulsive, paranoid, dependent, and avoidant) than in others, and Cluster C diagnoses became as prevalent as Cluster B diagnoses. In general, the differences between patients with and without PDs decreased, but there were still statistically significant differences concerning age, education, employment, number of SUDs, and scores on the SCL-90-R, GAF-S, and GAF-F.

## Discussion

### Prevalence of PDs

The main finding of this study was that about half of the sample of patients admitted for the first time with SUDs had a comorbid PD. In most earlier studies of clinical samples, the prevalence has been estimated to be around 60% [4]. A previous Norwegian study found a 72% prevalence of PDs in SUD patients [34]. This study applied the Millon Clinical Multiaxial Inventory (MCMI-II), a self-report diagnostic inventory, for assessment of Axis II diagnoses. The MCMI-II may overestimate the prevalence of PD diagnoses in SUD patients [35]. The differences between studies may also be explained by our inclusion of patients at their first admission only. In studies of samples that include patients with longer treatment histories with several readmissions to treatment, a higher proportion of patients are likely to be more severely and chronically ill. Additionally, in those patients, the risk of including substance-induced symptoms, especially in antisocial or borderline PDs, is higher [9]. Our findings may be closer to a ‘true’ prevalence, as we included all treatment seekers from the catchment area and all treatment modalities, applied reliable diagnostic interviews, and controlled for other Axis I and somatic comorbidities.

### Prevalence of subtypes

Consistent with most other studies, we found that Cluster B PDs were the most prevalent in SUD patients [11,13,17]. However, the difference between Cluster B and Cluster C diagnoses in our study was not statistically significant. When we included subthreshold diagnoses, Cluster C diagnoses were numerically more prevalent. Some studies have found PDs other than antisocial PD and borderline PD to be most prevalent. This may be explained by sample selection and assessment methods.

The prevalence of PDs in SUD patients is far above that found in epidemiological studies of PDs. A Norwegian study from the capital, Oslo, found PDs in 13.4% of the population [36] compared with 46% in our clinical sample. Other studies have found even lower rates in the general population [37,38]. The selection bias of hospital wards results in a higher prevalence of comorbidity in clinical samples. This is often referred to as Berkson’s fallacy [39]. When we compared the different PD diagnoses in our sample with those of a community sample, we found a particularly high prevalence of antisocial (16.4% versus 0.7%), borderline (13.1% versus 0.7%), paranoid (8.2% versus 2.4%), obsessive-compulsive (8.2% versus 2.0%), and avoidant (8.2% versus 5.0%) PDs. The prevalence pattern of PDs in SUD patients is different from the PD pattern found in patients with other Axis I diagnoses. In patients with mood or anxiety disorders, obsessive-compulsive, paranoid, and avoidant PDs are the most prevalent, while antisocial PD is far less prevalent than in SUD patients [40]. Interestingly, accidentally injured patients admitted to a surgical department had prevalence rates more similar to SUD patients [41].

### Axis I comorbidity

Patients with SUDs and PDs had a higher number of additional Axis I disorders than SUD patients without PDs. The low GAF-S scores and the high scores on the SCL-90-R and IDS indicated higher symptom loads in patients with PDs. Mood disorders were prevalent, but there were no differences between the groups. Previous studies have found more depression in SUD patients with PDs than in those without PDs [11,18]. Those studies assessed depressive symptoms in substance users without using diagnostic interviews reliability-tested in patients with comorbid disorders. Consistent with other studies [18], we found higher depressive symptom measures in PD patients, but we found no significant difference in the prevalence of MDD.

Anxiety disorders were more prevalent in SUD patients with PDs than in those without PDs in our study, consistent with previous studies. Almost 70% of the SUD patients with PDs had an anxiety disorder, most often social phobia. The high prevalence of social phobia in SUD patients [42] and the high prevalence of SUDs in patients with social phobia [43] indicate that patients with this disorder may have a disposition towards excessive substance use. The mean age at onset of social phobia was 10.4 years in the patients with PD and 13.2 years in the patients without PDs. The mean age of onset of SUDs in these two groups was 20.4 and 41.0 years, respectively, a difference that was statistically significant (p = 0.018). The combination of social phobia and PD seems to increase the risk of early onset SUD.

### Demographics and functioning

In our study, male and female SUD patients had the same overall prevalence of PDs. This is consistent with the previous Norwegian study of Landheim et al. [34]. We found antisocial PD in almost a quarter of the male patients, but in no female patients. However, Landheim et al. found the same prevalence of antisocial PD in women and in men (28% and 32%). In the general population in Oslo, Norway, PDs were found in 12.6% of the women and 13.7% of the men (the difference was not statistically significant) [36]. Consistent with our study, they did not find antisocial PD in women. This community study found statistically significant differences between women and men in the prevalence of antisocial PD (0% versus 1.3%), obsessive-compulsive PD (1.3% versus 2.6%), and passive-aggressive PD (0.9% versus 2.2%). Other studies have also provided inconsistent findings. In a sample of drug-dependent patients, Kokkevi et al. found no statistically significant differences between genders on any PD diagnoses [13]. In a sample of opioid abusers, Brooner et al. found that women were less likely than men to have a PD, twice as many males as females had antisocial PD, and seven times as many females as males had borderline PD [11]. These inconsistent findings are probably explained by sample selection. In addition, the very high prevalence of antisocial behavior in the most severely dependent substance users may be a result of living with addiction, and not a primary PD.

Patients with SUDs and comorbid PDs were less likely to attend school or work. The lower GAF-F scores indicated a lower level of functioning in the PD group. Our finding of more functional impairment in SUD patients with PDs is consistent with the findings of previous studies [4,18]. The decrease in differences between patients with and without PDs when subthreshold diagnoses are included implies, as expected, that the patients with subthreshold diagnoses have somewhat better functioning and fewer comorbid disorders than patients who meet the full criteria.

### Age at onset and early intervention

SUD patients with PDs were admitted to treatment for the first time at an earlier age than SUD patients without PDs. Patients with PDs developed their first SUDs at an earlier age, used more substances, and were heavier users of illicit substances. Childhood personality traits that make subjects susceptible to substance use, such as novelty seeking and disinhibition [44], may cause problems with resisting illicit substances and restricting the quantity of substances, including nicotine.

Of all the main Axis I diagnostic categories, the anxiety disorders had the earliest age at onset (attention deficit/hyperactivity disorder excluded), consistent with the findings of other studies [45]. It has been hypothesized that anxiety and affective symptoms can be partial mediators between childhood trauma and SUDs [46].

Even with the heavy burden of symptoms and impairment at a young age, patients with SUDs and PDs had their SUDs for several years more than patients without PDs before seeking treatment. SUD patients with comorbid PDs develop their disorders before their nervous system has matured [47], and before they have had experience in coping with adversities and life problems. This explanation contributes to the understanding of the poor prognosis. Conduct disorder in childhood is a precursor of antisocial PD. Conrod et al. found that personality-targeted interventions in high-risk young adolescents delayed the onset of drinking and binge drinking [48]. Early intervention, focusing on personality traits and substance use in young adolescents with conduct disorder, may restrict the progression into PDs and SUDs.

Outcome studies of samples of SUD patients have shown that the co-occurrence of PDs has a high negative predictive value for future abstinence in both AUD and DUD patients [1,4,49,50]. It has been hypothesized that this is related to the PD patients’ lower ability to form treatment alliances [51] or their lower ability to cope with negative affects [52]. The combination of comorbid Axis I and Axis II disorders in SUD patients makes the outcome of the SUDs even more adverse [53,54]. In addition, the outcome of the PD is worse if the SUD is not treated [55]. In summary, our findings emphasize the necessity of diagnosing comorbid Axis I and Axis II disorders in SUD patients in clinical practice.

### Strengths and limitations

The strengths of this study lie in the sample collection and assessment methods. The catchment-area-based services made it possible to identify all patients who met the study criteria. Other specialized addiction or psychiatric services, which received patients from the catchment area, cooperated by identifying eligible patients and referring them to the study. In contrast to earlier studies, the PDs in our sample were assessed at a relatively early stage. By selecting a sample of patients at their first admission, we avoided an overrepresentation of the chronically ill, and we reduced recall bias. Furthermore, we obtained reliable assessment of all common SUDs, Axis I, and Axis II disorders, by using reliability-tested diagnostic interviews performed by a psychiatrist. The SCID-II was chosen for its good criterion validity and reliability in diagnosing PDs. The PRISM is the best-documented diagnostic interview for diagnosing a wide range of Axis I disorders in heavy substance users. The use of different methods to assess some of the same symptom areas showed consistent results. This strengthens the findings. SUD patients are at high risk of noncompliance. Even so, we had a low dropout rate of four out of 78 (5%), which was achieved by the personal follow-up of each patient.

The limitations of this study lie in the relatively small sample size, and the risk of type II errors. On the other hand, the statistically significant differences found when comparing small groups are reliable and interesting. The small sample size is due to the rigid inclusion rules: a single catchment area and the use of patients on their first admission only. Choosing a larger catchment area would have increased the sample size, but at the cost of losing the overview of all eligible patients. Some of the patients were using psychoactive substances during the interviewing period; there was no defined period of abstinence before the assessments. This may have influenced the patients’ statements and memory. However, the patients were interviewed in a stable phase, each patient was seen at several appointments, and inconsistencies were attended to. We did not account for multiple testing.

## Conclusions

Almost half of this representative sample of SUD patients, admitted for treatment for the first time, had PDs. PD assessment of SUD patients should not be limited to antisocial and borderline PD. SUD patients with PDs differ from those without PDs. They have a lower age at admission, a lower age at onset of their first SUD, more use of illicit drugs, more anxiety disorders, lower ability to attend work or school, more distressing symptoms, and more functional impairment. Thus, SUD patients with PDs have different treatment needs than SUD patients without PDs. SUD patients should therefore be thoroughly assessed for comorbid Axis I and Axis II disorders.

## Abbreviations

AUD: Alcohol use disorder; AUDIT: Alcohol use disorders identification test; DSM-IV/5: Diagnostic and statistical manual of mental disorders fourth/fifth edition; DUD: Drug use disorder; DUDIT: Drug use disorders identification test; GAF-F: Global assessment of functioning - functioning scale; GAF-S: Global assessment of functioning - symptom scale; GSI: Global symptom index (measured with SCL-90-R); ICD-10: International classification of disorders tenth edition; IDS: Inventory of depressive symptoms; MCMI-II: Millon clinical multiaxial inventory; MDD: Major depressive disorder; NOS: Not otherwise specified; PD: Personality disorder; PRISM: Psychiatric research interview for substance and mental disorders; PTSD: Post-traumatic stress disorder; SCID II: Structured clinical interview for DSM-IV Axis II personality disorders; SCL-90-R: Symptom checklist 90 questions, revised version; Sign.: Statistical significance; SUD: Substance use disorder.

## Competing interests

The authors declare that they have no competing interests.

## Authors' contributions

UFM organized and secured the financial support for the study. All authors contributed to the background, design, and drafting of the manuscript. All authors have read and approved the final manuscript.

## Pre-publication history

The pre-publication history for this paper can be accessed here:

http://www.biomedcentral.com/1471-244X/12/180/prepub
